# Comparison of lumbar plexus block using the short axis in-plane method at the plane of the transverse process and at the articular process: a randomized controlled trial

**DOI:** 10.1186/s12871-018-0480-1

**Published:** 2018-02-07

**Authors:** Rui Lu, Chengcheng Shen, Chunyong Yang, Yan Chen, Juanjuan Li, Kaizhi Lu

**Affiliations:** Department of Anesthesia, the First Hospital Affiliated to Army Medical University (Southwest Hospital), Army Medical University, Chongqing, 400038 China

**Keywords:** Regional anesthesia, Lumbosacral plexus, Epidural anesthesia

## Abstract

**Background:**

Although the safety and effectiveness of the short-axis in-plane method has been confirmed for lumbar plexus block, the operation is difficult and has a high rate of epidural spread at the plane of the articular process. Therefore, we developed a new in-plane technique, called the beach chair method, which displays images from the transverse process. We compared the operative difficulty and incidence of epidural spread of the beach chair method with those of the control method (at the plane of the articular process) in this randomized controlled clinical trial.

**Methods:**

Sixty patients, aged 18 to 75 years, scheduled for unilateral arthroscopic knee surgery were randomized to receive double-guided lumbar plexus block by the beach chair method (*n* = 30) or the control method (*n* = 30) with 30 ml 0.5% ropivacaine hydrochloride; all patients received a sciatic nerve block with 10 ml 1% lidocaine hydrochloride and 10 ml 0.5% ropivacaine hydrochloride.

**Results:**

The incidence of epidural spread after lumbar plexus block was significantly lower in the beach chair group than that in the control group [1 case (3.3%) vs. 9 (30.0%), *P* = 0.006]. Moreover, the imaging time (34.2 ± 16.7 s vs. 48.9 ± 16.8 s, *P* = 0.001), needling time (85.0 ± 45.3 s vs. 131.4 ± 88.2 s, *P* = 0.013) and number of needle punctures (2.7 ± 1.3 vs. 4.5 ± 2.1, *P* = 0.000) were significantly lower in the beach chair group than those in the control group; the ultrasound visibility score of the beach chair group was better than that of the control group. There were no significant differences in the remaining indicators.

**Conclusions:**

The beach chair method was easier and was associated with a lower incidence of epidural spread than the control method. Therefore, the beach chair method (at the plane of the transverse process) provides another promising option for lumbar plexus block for the non-obese population.

**Trial registration:**

Chinese Clinical Trial Registry (ChiCTR), Registration number:ChiCTR-INR-15007505, registered on November 06, 2015.

## Background

The lumbar plexus (LP) originates from T12 to L5. The obturator nerve is supplied by the anterior branch of L2-L4, and the femoral nerve is supplied by the posterior branch of L2-L4. The LP emanates from the intervertebral foramen and then penetrates down into the psoas major muscle inward and is located between the anterior two-thirds and the posterior one-third of the psoas major muscle. [1, 2] Lumbar plexus block (LPB) is a method of injecting liquid local anesthetic around the LP nerve, which innervates the front, medial and lateral aspects of the thigh. [3] Although spinal anesthesia completely blocks the afferentiation of the lower limb sensory nerve, the largest advantage of LPB is that it is a peripheral nerve block with few hemodynamic effects. [4] LPB anesthesia is an effective but not fully utilized regional block technique. [5] Several complications such as epidural spread often occur in pure-landmark LPB technology. [6–8]

The LP is positioned deep within the torso. [9, 10] The traditional posterior puncture approach [11, 12] with real-time ultrasound guidance is not easy to perform in clinical practice, and there has been no comparative study of LPB techniques to determine which puncture technique is most suitable for ultrasound guidance. [13] Currently, the short-axis in-plane method is commonly used for in-plane ultrasound-guided LPB in clinical practice; however, in the control method (at the plane of the articular process) [14], the puncture site is close to the bony structure, which is located on the midline of the back, and the puncture angle could be limited by the obscuration of the articular process. Thus, the length of the ultrasound image of the LP root could be very short (The typical strip shaped high-echo image could not be developed easily), which would increase the difficulty of the operation. Therefore, we developed a new LPB method called the beach chair method that utilizes an innovative puncture passage and a different imaging plane (at the plane of the transverse process). In this study, we compared the beach chair method with a control method in terms of technique difficulty and incidence of epidural spread.

## Methods

This study [Protocol no.: Scientific Research No. (62) of 2015] was approved by the Ethics Committee of the Southwest Hospital affiliated (the First Affiliated Hospital) to Third Military Medical University, Chongqing, China on November 06, 2015 and has been registered at http://www.chictr.org.cn (ChiCTR-INR-15007505). The trial was conducted in accordance with the Declaration of Helsinki and monitored by the Good Clinical Practice (GCP) unit at the Southwest Hospital of Third Military Medical University.

The patients in this study were among those who were scheduled for arthroscopic unilateral knee joint surgery between November 2015 and September 2016 at Southwest Hospital. Inclusion criteria were males and females aged 18 to 75 years and patients who volunteered to participate in the study and signed informed consent. Exclusion criteria were emergency surgery, American Society of Anesthesiologists (ASA) Class IV or V, pregnancy or lactation, recent use of anticoagulant drugs (heparin) or antiplatelet drugs (aspirin), allergy to local anesthetics, infection at the puncture site, and neurological disorders or significant difficulties in normal communication (hearing, vision, intelligence or mental abnormalities).

Patients who met the inclusion criteria received written information about the trial and signed their consent at their first consultation in the anesthesia outpatient clinics. The patients were then assigned a random number (ranging from 1 to 60, without repeats), which was generated by a computer-generated randomization code. Each random number was placed in a sealed envelope by an uninvolved third person, and patients received an envelope according to their order of visit in the anesthesia outpatient clinics. We decided to assign odd-numbered patients to the beach chair group and even-numbered patients to the control group. The LPB was performed by an anesthesiologist (CY, attending doctor) who received training in regional block anesthesia for 3 years and was familiar with many methods, including LPB and quadratus lumborum block. The operator was informed of the patient grouping information, but postoperative evaluators were not allowed to enter the block room during the operation to prevent leakage of the patient group information. The group allocation was disclosed to the participants after all the assessments were finished.

Patients were taken to the block room, where heart rate (HR), blood pressure (BP), pulse oxygen saturation (SpO_2_) and electrocardiogram (ECG) data were monitored continuously with a Solar 8000 monitor. After intravenous access was established, 1 mg midazolam and 50 μg fentanyl citrate injection were injected intravenously. Patients were placed in a lateral position with the ipsilateral leg up. The skin from the ipsilateral trunk to the upper thigh was disinfected with iodophor and covered with a sterile towel. Scanning was performed with a Hivision Preirus system (Hitachi Medical Co., Ltd.) using a convex array probe and a frequency of 2–5 MHz. The probe was coated with the coupling agent and covered with a sterile guard. For the sciatic nerve block [15], the operator performed the puncture combined with nerve stimulation (settings: current 1.0 mA, frequency 1 Hz) in a sterile manner as follows: The intermediate point between the greater trochanter of the femur and the posterior superior iliac spine (PSIS) was marked, and the probe was then placed on the connection between this point and the PSIS. At this point, the high-echo image of the ilium was seen on ultrasound, and the probe was then moved toward the tail end and in an inward direction until the ultrasound image of the sacrum and ilium appeared at the same time. The elliptic high-echo image between the sacrum and the ilium indicated the sacral plexus. The probe was then slid to position the image of the sacral plexus in the middle of the screen; the puncture was made at the middle of the probe by using the short-axis in-plane method with an 11 cm needle. When contraction of the gastrocnemius occurred at a current of 0.3 mA, 10 ml 1% lidocaine hydrochloride and 10 ml 0.5% ropivacaine hydrochloride were injected. After the sciatic nerve was blocked, LPB was performed as follows.

### Control method (at the plane of the articular process) details (Fig. 1)

With the patient in the lateral position, the low-frequency convex probe was placed on the paravertebral line and parallel to the spine. The probe was then slid from the caudal end to the cranial end to confirm the gap position between L3 and L4; the probe was then rotated 90 degrees at that position. The depth was recorded when the transverse process appeared on the screen. Subsequently, the probe was slid slightly toward the head end or the tail end in an effort to avoid the transverse process and to show the images of the articular process, spinous process and LP root. The puncture site was located 1 cm outside the long axis of the ultrasonic probe. After the patient received local anesthesia, the needle was moved toward the downward direction of the articular process until a quadriceps twitch was triggered, and then the current of nerve stimulator was gradually reduced to 0.3–0.5 mA. If the quadriceps continued to twitch, it was safe to assume that the needle was close to the LP; 30 ml 0.5% ropivacaine hydrochloride was then injected, confirming that no blood was present. The operator recorded the imaging time (time from probe placement until the puncture needle was inserted into the skin), ultrasound visibility score (UVS) [16] (visibility of the paravertebral structures in the sonograms was assessed by using a 4-point Likert scale: 0, not visible; 1, hardly visible; 2, well visible; 3, very well visible), needling time (time from needle insertion until the drug was injected) and the number of needle punctures. After injection, the evaluators assessed the knee joint sensory and motor status and relevant complications.

**Fig. 1:**
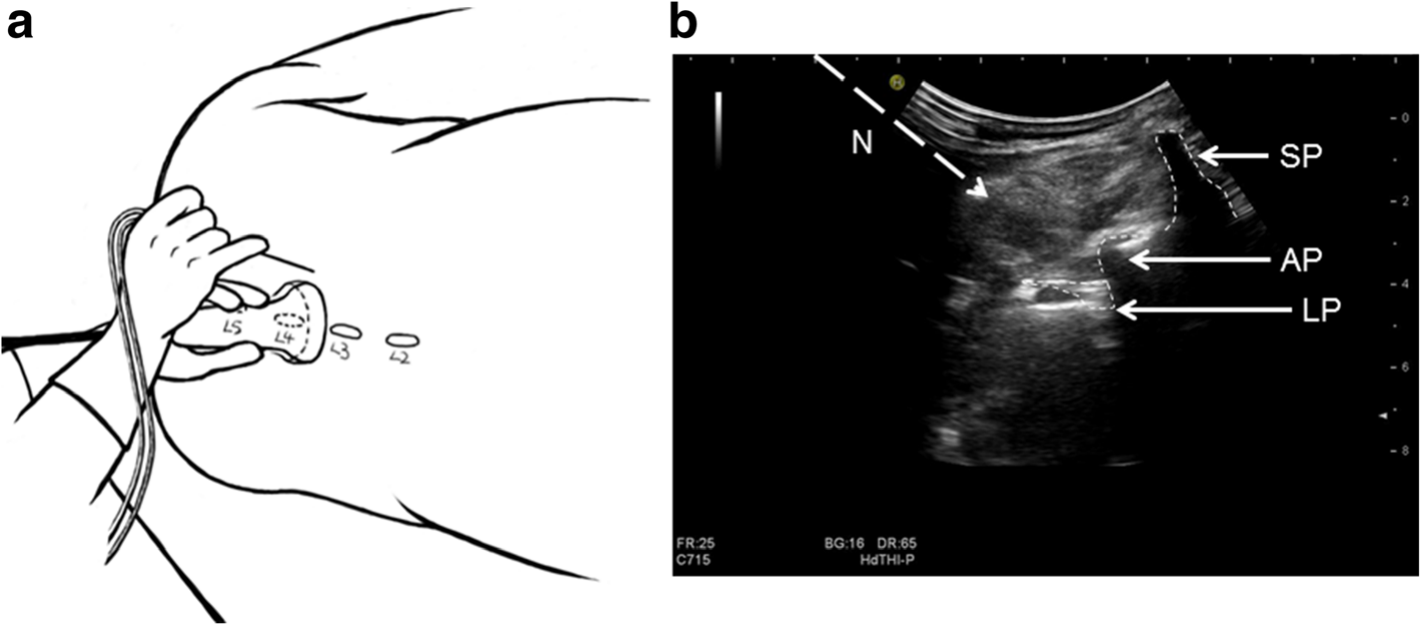
**a** Schematic diagram of the probe placement and the puncture site in the control method. **b** Ultrasound image and puncture passage of the control method. SP, spinous processes; AP, articular processes; LP, lumbar plexus; N, needle

### Beach chair method (at the plane of transverse process) details (Fig. 2)

The patient was in the lateral position. The low-frequency convex probe was placed transversely on the lateral abdomen and at the top of the iliac crest. The ultrasound showed a “beach chair”-shaped continuous ultrasonic signal, which was formed by the ultrasonic signal of the transverse process, pedicle and the lateral edge of vertebral body. A “rainbow”-shaped high-echo signal (LP root) across the posterior quarter quadrant of the psoas major was also displayed on the screen. The needle was then inserted at 1 cm next to the long axis of probe. After the patient received local anesthesia, the needle was moved close to the transverse process and advanced in the lateral direction of the vertebral body until reaching the posterior quarter quadrant of the psoas major muscle, which was close to the similar “rainbow”-shaped ultrasonic signal (LP root). Local anesthetic (30 ml 0.5% ropivacaine hydrochloride) was injected when the quadriceps twitch was triggered as described above in the control method. The operator recorded the imaging time, UVS, needling time and number of needle punctures. After injection, the evaluators assessed the knee joint sensory and motor status and relevant complications.

**Fig. 2:**
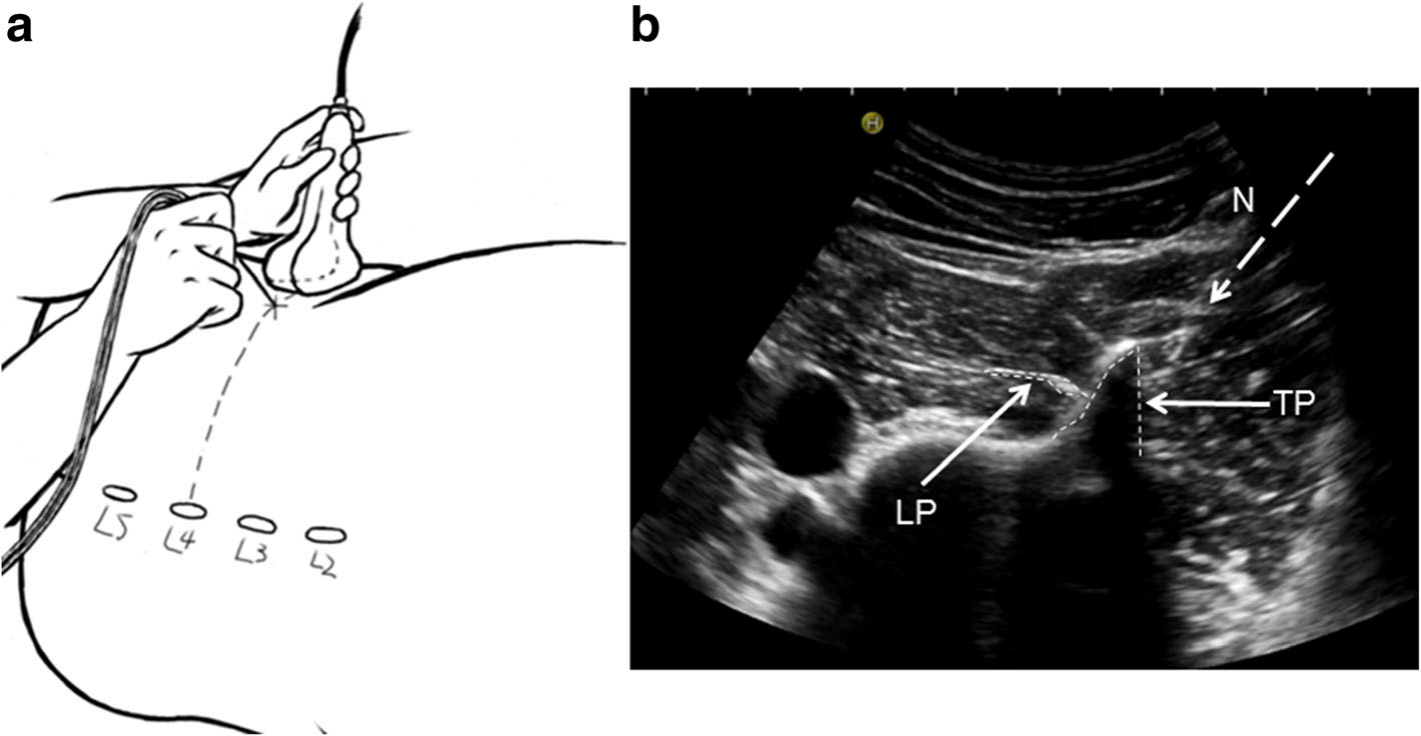
**a** Schematic diagram of the probe placement and the puncture site in the beach chair method. **b** Ultrasound image and puncture passage of the beach chair method. TP, transverse process; LP, lumbar plexus; N, needle

### Assessment after block

LPB was considered successful when the quadriceps twitch was triggered at a current of 0.3–0.5 mA and local anesthetic was injected successfully. A blinded study assistant assessed the sensory and motor status of both knee joints 5, 15, and 30 min after drug injection; the sensory status of the knee joint was determined by assessing the main dominant nerves, including the femoral nerve, the lateral femoral cutaneous nerve and the obturator nerve. Sensation was assessed by both ice application and a pinprick test. Ice application was used to assess temperature sensation, and the pinprick test was used to assess pain and tactile sensation. The degree of sensory block was assessed with 4 levels: 0 (no block); 1 (decreased temperature sensation); 2 (no temperature sensation and no pain sensation but normal tactile sensation); and 3 (no temperature or pain sensation). A score greater than or equal to 2 was considered an effective sensory block. An obturator block was considered successful when both sensation and thigh adduction were blocked. Therefore, we combined the sensory assessment and motor assessment of the obturator nerve in our study. We considered that epidural anesthesia occured when a sensory block was present on both sides of the leg. The motor block degree was assessed by an improved 4 levels scale as described before [17]: 0 (no motor function); 1 (unable to move against resistance); 2 (able to move against resistance but decreased); and 3 (normal strength). A score less than or equal to 2 was considered a successful motor block. VAS pain rating was assessed in the post-anesthesia care unit (PACU) 24 h after the LPB. For the pain assessment, patients were asked to make a mark on a 10-cm line corresponding to their pain level, with “0” being “no pain at all” and “10” being “the worst pain”.

### Statistics analysis

This trial design was superiority and the sample size was calculated based on the results of a preliminary experiment, which found that the incidence of epidural spread (the primary outcome) in the beach chair method was 6.67% and that of the control method was 40%. We defined the α value as 0.05 and β as 0.1. The delta for sample size calculation was 0.33. The sample size was 30 cases for each group, which was calculated using the sample estimation software PASS 11.0. Statistical analysis was performed using SPSS 13.0. The count data were analyzed by the χ^2^ test, and the rank data were analyzed by the Wilcoxon rank sum test. The minimum expected count of epidural spread incidence analysis was 5; we used the Pearson chi-square test. The two independent samples, which were normally distributed, were measured by the t test, and the non-normally distributed data were measured by the Mann-Whitney U test. *P* < 0.05 was considered statistically significant.

## Results

**Fig. 3:**
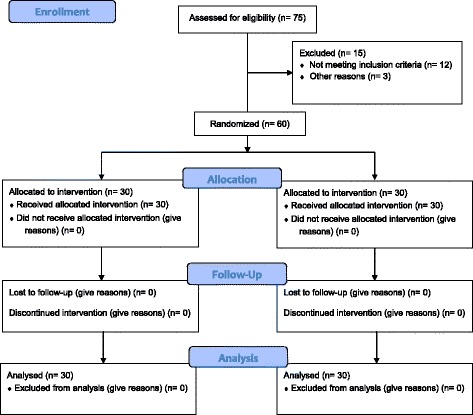
CONSORT diagram

Seventy-five patients were screened from July 2015 to September 2016. Among these, 12 patients were excluded because their age did not meet the inclusion criteria, and 3 patients were excluded because they required bilateral knee surgery. Ultimately, 60 patients were included in this study, 30 in each group; all patients completed the process according to the research protocol (Fig. 3). The demographic and perioperative data are summarized in Table 1.

**Table 1:** Patient demographic and perioperative data

Variables	Beach chair	Control
	(*n* = 30)	(*n* = 30)
Gender, M:F	15/15	11/19
Age, y	40.2 ± 13.7	44.8 ± 11.5
Height, cm	163.6 ± 8.5	159.9 ± 7.6
Weight, kg	64.4 ± 11.8	62.1 ± 10.1
BMI, Kg/m	24.1 ± 4.0	24.2 ± 2.6
ASA classification, I/II	9/21	10/20
Surgery time, min	81.6 ± 37.7	63.9 ± 33.7
Surgery Type		
Meniscal repair	16(53.3)	19(63.3)
Subtotal meniscus resection	5(16.7)	3(10.0)
Knee cavity laparotomy	3(10.0)	2(6.7)
Popliteal cyst resection	1(3.3)	1(3.3)
Joint free body removal	0(0)	3(10.0)
Cruciate ligament reconstruction	5(16.7)	2(6.7)

Values are presented as Mean ± SD or No. (%)

The imaging time (34.2 ± 16.7 s vs. 48.9 ± 16.8 s, *P* = 0.001), the needling time (85.0 ± 45.3 s vs. 131.4 ± 88.2 s, *P* = 0.013) and the number of needle punctures (2.7 ± 1.3 vs. 4.5 ± 2.1, *P* = 0.000) of the beach chair group were significantly less than those of the control group, while the UVS of the beach chair group was better than that of the control group (*P* = 0.000; Table 2). The incidence of epidural spread in the beach chair group was significantly lower than that in the control group [1 (3.3%) vs. 9 (30.0%), *P* = 0.006; Table 2]; there was no difference in the 24-h VAS scores between the two groups.

**Table 2:** Nerve block procedure, anesthesia recovery time, epidural spread incidence and pain ratings

Variables	Beach chair	Control	*P*
	(n = 30)	(n = 30)	
Imaging time, sec	34.2 ± 16.7	48.9 ± 16.8	0.001^*^
Needling time, sec	85.0 ± 45.3	131.4 ± 88.2	0.013^*^
Ultrasound visibility score, 0/1/2/3	0/0/3/27	0/2/14/14	0.000^*^
Number of needle punctures^a^	2.5(2)	4(1)	0.000^*^
Sensory block time, hour	11.9 ± 5.4	11.4 ± 5.9	0.717
Motor block time, hour	18.1 ± 5.3	16.0 ± 6.0	0.167
Epidural spread incidence	1(3.3)	9(30.0)	0.006^*^
24 h VAS scores	3.7 ± 2.0	4.1 ± 2.0	0.372

Values are presented as Mean ± SD or No. (%)

Scores 0, 1, 2, and 3 correspond to not visible, hardly visible, well visible, very well visible

^a^Values are presented as Median (IQR)

^*^Statistically significant

All patients in both groups had a successful LPB. The patients who involved in this study did not receive a general anesthetic or spinal anesthetic but mild sedation (1 mg midazolam after intravenous access was established). The anesthesia effect in both groups was good, and remifentanil was not used during surgery. There was no significant difference in the ipsilateral sensation and the motor block rate between the groups at 5 min, 15 min and 30 min after the LPB. There was also no significant difference in knee joint sensation and motor blocking time. No complications occurred in either group.

## Discussion

Dual guidance provides not only an imaging reference but also a reference for regional motor reflexes. As for beach chair method, the possibility of injury to bowel during the technique does exist because of its lateral needle path; however, the possibility of this complication could be minimized if we keep needling in the right path which is close to the posterior edge of quadratus lumborum and anterior edge of transverse process. In this study, we compared the beach chair method (at the plane of the transverse process) and the control method (at the plane of the articular process) for LPB and found that the incidence of epidural spread in the beach chair group was significantly lower than that in the control group. Moreover, we found that the UVS of the beach chair group was better than that of the control group. Finally, the operation difficulty indicators of the beach chair method, including the imaging time, the needling time and the number of needle punctures, were less than that of the control group. It is possible that a prolonged operating time may cause discomfort, such as the discomfort of maintaining a fixed position for a long time and puncture discomfort. Reducing both positioning time and puncture time is certainly beneficial for the patient.

Previous studies have shown that LPB may lead to epidural spread. Philippe Biboulet et al. reported that the rate of epidural spread was 26.7% using the nerve stimulator-guided Dekrey L3 method, which is similar to the result of the control group in our study. [18] Biboulet explained that the drug may infiltrate the epidural space from the intervertebral foramen when injected in the paravertebral region. [19, 20] This explanation suggests that intraspinal drug infiltration could be avoided to some extent by injecting the drug away from the intervertebral foramen. To date, there has been no report on the incidence of epidural spread in the double-guided short-axis in-plane method. In our study, we found that the incidence of epidural spread for the control method was significantly higher than that for the beach chair method. We propose four reasons for this difference. (1) The puncture approach of the control method is similar to that of the Dekrey L3 method. (2) The position of the puncture needle near the nerve root in the beach chair method is further outside than in the control method. Thus, the tangent plane of the puncture is not the same as the intervertebral foramen because the “beach chair” image is a continuous image. For the control method, the epidural spread incidence is very high because the articular process is adjacent to the intervertebral foramen, and the site of drug injection is at the lateral aspect of the intervertebral foramen. (3) The beach chair method is performed in the transverse plane, and its puncture needle is away from the intervertebral foramen and blood vessels, which may also be a reason for the reduced incidence of epidural spread. (4) In the beach chair method, the positional relationship between the needle tip, injected drug, psoas major, intervertebral foramen and the lateral edge of vertebral body can be observed, enabling tracking of approximate diffusion range of the injected drug by skewing the probe slightly [21]; this relationship could not be observed in the control method because of its poor deep imaging quality.

We also analyzed some of the reasons why the beach chair method is more advantageous than the control method in terms of operation and ultrasound imaging. The beach chair method allows the lumbar region to be juxtaposed at the same depth and then imaged at the same time, so the method can show more areas for the puncture. Furthermore, the beach chair method is more convenient to perform because its puncture passage has no bony structures to block it and the operator’s hand remains close to the ultrasonic probe, which could allow locating the needle in-plane and continuous visualization more easily. For the control method [14], the operator’s hand is close to the posterior aspect of the articular process so that the ultrasound signal of the needle could be blocked by the articular process when it is close to the nerve root. The shamrock method, which was first described by Sauter AR, is a useful technique, and our method is similar except for the site of needle insertion [22]; however, the ultrasound signal of the needle is often blocked by the transverse processes in the shamrock method because of its posterior puncture direction, which causes the puncture needle to skew out of the ultrasonic plane (Puncture needle deflected and is not in the ultrasonic plane). Although the shamrock method, which was improved by Lin [23], adopted a new puncture approach through the inter-transverse process, its main puncture site is located in the lateral intervertebral foramen, which may lead to intraspinal drug infiltration.

Undoubtedly, our research has some limitations. First, we did not encounter a particularly obese patient in our study; therefore, the utility of the beach chair method for obese patients will require further study. Furthermore, we did not compare the shamrock method with the beach chair method in this study; this comparison will be made in the next study.

## Conclusion

The beach chair method, which adopts an innovative lateral puncture approach method, has advantages over the control method in terms of the imaging time, the ultrasonic imaging quality, the needling time and the number of needle punctures. This method also provides another LPB option for patients with puncture limitations (such as posterior back infection and inability to change position). Compared with the control method, the beach chair method had a significantly lower incidence of epidural spread, which could ensure more accurate unilateral anesthesia. These results suggest that the beach chair method could become another promising technical choice for LPB.

## Abbreviations

ASA: American Society of Anesthesiologists
LFCN: Lateral femoral cutaneous nerve
LP: Lumbar plexus
LPB: Lumbar plexus block
UVS: Ultrasound visibility score
